# Identification of risk areas and associated factors of unfavorable treatment outcomes in drug-resistant tuberculosis: Evidence from Rio de Janeiro, Brazil

**DOI:** 10.1371/journal.pone.0348788

**Published:** 2026-05-08

**Authors:** Heitor Levy Ferreira Praça, Juliana Cavalcanti Figueiredo, Natalia Santana Paiva, Jefferson Pereira Caldas dos Santos, Paulo Victor de Sousa Viana, Rejane Sobrino Pinheiro, Antonio Jose Leal Costa, Gerusa Gibson, Alexandre San Pedro

**Affiliations:** 1 Instituto de Estudos em Saúde Coletiva, Universidade Federal do Rio de Janeiro – UFRJ, Rio de Janeiro, Brasil‌‌; 2 Laboratório e Inteligência Geográfica em Ambiente e Saúde, Universidade Veiga de Almeida, Rio de Janeiro, Brasil; 3 Instituto de Tecnologia em Fármacos, Fundação Oswaldo Cruz, Rio de Janeiro, Brasil; 4 Centro de Referência Professor Hélio Fraga, Fundação Oswaldo Cruz, Rio de Janeiro, Brasil; Federal University of Ceara, BRAZIL

## Abstract

Drug-resistant tuberculosis remains a major challenge to tuberculosis control in Brazil and worldwide.

**Objective:**

To identify factors associated with unfavorable treatment outcomes and to analyze the occurrence of high-risk spatial clusters at an intra-urban scale in the municipality of Rio de Janeiro, Brazil.

**Methods:**

A retrospective cohort study of drug-resistant tuberculosis cases reported between 2015 and 2022 was conducted. Individual-level and spatial analyses were performed to identify associated factors with unfavorable outcomes and high-risk clusters.

**Results:**

Of the 972 cases analyzed, 31.6% had unfavorable outcomes, including 20.2% lost to follow-up, 4.9% with treatment failure, and 6.3% deaths. Loss to follow-up was higher among males, Black individuals, those with low education levels, individuals with AIDS, and those who used alcohol, tobacco, or illicit drugs, whereas older age and diabetes were associated with lower odds. Treatment failure was more frequent among individuals of Brown race/skin color, those with multidrug-resistant and extensively drug-resistant tuberculosis, and those with multiple previous treatments. Mortality was higher among individuals over 40 years of age, those with multidrug-resistant and extensively drug-resistant tuberculosis, those with alcohol use, and individuals AIDS. Three high-risk spatial clusters were identified in the northern zone of the city, an area of marked social vulnerability.

**Conclusion:**

Unfavorable drug-resistant tuberculosis outcomes are influenced by socioeconomic factors, particularly in socially vulnerable urban areas. In this context, strengthening local surveillance in identified high-risk clusters, improving patient-centered care across primary and secondary levels, and providing robust socioeconomic support are essential strategies for reducing unfavorable outcomes and achieving more equitable tuberculosis control.

## Introduction

Declared a global health emergency by the World Health Organization (WHO) in 1993, tuberculosis remains one of the most challenging communicable diseases for public health worldwide. Reducing its morbidity and mortality rates is a key target in global sustainable development agendas, as highlighted by international frameworks such as those of the United Nations [1] and WHO [2].

The interruption of treatment, the presence of comorbidities among affected individuals, and systemic challenges in implementing timely and comprehensive drug susceptibility testing have collectively contributed to the emergence and persistence of drug-resistant forms of tuberculosis [3–5].

In Brazil, where tuberculosis has historically posed a significant challenge to the Unified Health System (SUS), the emergence of drug-resistant tuberculosis (DR-TB) cases to first-line medications has further limited the effectiveness of disease control efforts [6]. This is particularly concerning as the burden of DR-TB falls most heavily on populations and groups in situations of greater social vulnerability [7,8]. In the national context, tuberculosis serves as a marker of health inequities, disproportionately affecting populations in densely populated urban areas with precarious housing. The disease thus both reflects and perpetuates cycles of poverty, social exclusion, and unequal access to healthcare [9,10].

In the case of DR-TB, unfavorable outcomes represent particularly severe scenarios, involving both challenges in treatment management and individual biological conditions, which are often worsened by contexts of intense social vulnerability [3,8].

At the national level, the city of Rio de Janeiro stands out for its high incidence of both drug-susceptible tuberculosis and DR-TB. The spatial distribution of DR-TB indicates that cases of the resistant form have been reported in all Brazilian states, with Rio de Janeiro recording the highest burden. From 2015 to 2018, 613 cases were reported (15.5% of the national total), increasing to 675 (13.4%) from 2019 to 2023 [11]. Within the state, the capital accounted for 70.8% and 69.4% of all DR-TB cases in 2022 and 2023, respectively [12].

These statistics highlight the disproportionate burden of tuberculosis and DR-TB in Rio de Janeiro, underscoring the need for a deeper understanding of the social determinants and spatial dynamics driving this epidemic at the municipal level, where TB care and control interventions are primarily implemented.

Understanding the factors associated with these unfavorable outcomes and their intra-urban spatial distribution enables the identification of both affected populations and high-risk cluster areas. However, conventional analytical approaches often rely on regression models that assume independence of observations and homogeneous relationships across space, assumptions that ignore the principle of spatial dependence and rarely hold for drug-resistant tuberculosis, given its focal transmission driven by socio-environmental and healthcare-related conditions. Spatial scan statistics offer a robust alternative by explicitly testing for the presence of such dependence, identifying areas and periods of concentrated risk and estimating relative risks within detected clusters. This information is strategically important for surveillance, guiding targeted control actions in high-burden regions [13].

Considering the context of DR-TB emergence and dissemination in Brazil, this study aims to identify sociodemographic and epidemiological factors associated with unfavorable treatment outcomes in DR-TB cases and analyze the occurrence of high-risk spatial clusters in the city of Rio de Janeiro, Brazil.

## Methods

### Study design and data source

This study employed a retrospective cohort of drug-resistant tuberculosis cases among residents of the municipality of Rio de Janeiro (MRJ), reported in the Special Tuberculosis Treatment Information System (SITE-TB) between January 2015 and December 2022. The data was obtained for research purposes on 01/05/2024. Individual-level analyses identified factors associated with unfavorable treatment outcomes, while ecological analyses using a spatial approach at the neighborhood level identified high-risk clusters for these outcomes.

Since 1993, tuberculosis cases in Brazil have been subject to mandatory notification and recorded in the *Sistema de Informação de Agravos de Notificação* (Notifiable Diseases Information System, SINAN in Brazilian acronym) [14]. In 2012, a specific system for drug-resistant tuberculosis cases was introduced to strengthen surveillance and monitoring efforts: the *Sistema de Informação de Tratamentos Especiais da Tuberculose* (Special Tuberculosis Treatment Information System, SITE-TB) [15].

A tuberculosis case is registered in SITE-TB only after its closure in SINAN following a confirmed diagnosis of resistance to one or more first-line treatment drugs [16]. Healthcare professionals input data into SITE-TB, which is subsequently validated by physicians from tuberculosis reference units nationwide [17]. The system stores clinical and demographic data, including drug susceptibility test results, adverse events, treatment regimens, and treatment outcomes for each registered case [18].

The definitions for classifying treatment outcomes, types of resistance, and drug resistance patterns were applied following WHO guidelines:

- Treatment success is defined as when a patient is cured (bacteriologically confirmed negative at the end of treatment) or completes treatment without evidence of failure.
- Cases classified as lost to follow-up (treatment interruption) are those in which individuals discontinued treatment for two consecutive months or more.
- Treatment failure is defined as the persistence or worsening of a positive bacteriological result at the end of the treatment period, or the emergence of resistance amplification during therapy.
- TB-related death is defined as death where tuberculosis is the direct cause or significantly contributes to the fatal outcome before treatment completion.
- Primary resistance refers to cases of drug-resistant tuberculosis diagnosed in patients who have never been treated for tuberculosis or have received less than one month of prior treatment.
- Acquired resistance occurs when resistance develops in a patient previously treated for tuberculosis, indicating treatment failure or inadequate adherence to the drug regimen.
- Monoresistance refers to resistance to a single first-line anti-TB drug.
- Polyresistance refers to resistance to two or more first-line drugs, excluding both rifampicin and isoniazid.
- Rifampicin-resistant TB (RR-TB): caused by a strain of *M. tuberculosis* complex that is resistant to rifampicin.
- Multidrug-resistant tuberculosis (MDR-TB) refers to resistance to at least rifampicin and isoniazid, the two most potent first-line anti-TB drugs.
- Pre-extensively drug-resistant TB (pre-XDR-TB): caused by a strain of *M. tuberculosis* complex that is resistant to rifampicin, and that is also resistant to at least one fluoroquinolone.
- Extensively drug-resistant tuberculosis (XDR-TB) refers to MDR-TB cases that also exhibit resistance to fluoroquinolones and at least one second-line injectable drug (amikacin, capreomycin, or kanamycin).

Cases from outside the municipality of Rio de Janeiro were excluded to ensure geographic representativeness and analytical homogeneity in the assessment of treatment outcomes. Individuals under 20 years of age (8%) were excluded because this age group presents different clinical and therapeutic profiles, including different drug metabolism, age-specific dosing regimens, and distinct diagnostic approaches. Cases identified as Asian or Indigenous were excluded due to their small sample size of 0.26%, which precludes meaningful statistical inference and could introduce instability in association analyses. Additionally, cases with closure reason ‘change in diagnosis’ were excluded as they were not confirmed DR-TB cases, and cases transferred to another country or municipality were excluded because outcome information was not available for follow-up.

In January 2025, the Brazilian Ministry of Health incorporated pretomanid into the public healthcare system for the treatment of drug-resistant tuberculosis, aligning with the World Health Organization guidelines [19]. With this update, the BPaLM regimen (comprising bedaquiline, pretomanid, linezolid, and moxifloxacin) became the preferred first-line therapy for RR-TB, MDR-TB, pre-XDR-TB, and XDR-TB, with a six-month duration [20].

In cases of treatment failure with BPaLM, due to bacterial resistance or severe adverse effects, the prolonged regimen (18 months) is recommended, consisting of a 6-month intensive phase with bedaquiline, levofloxacin, linezolid, terizidone, and for MDR-TB/XDR-TB, the addition of injectable amikacin, followed by a 12-month continuation phase with levofloxacin, linezolid, and terizidone. The most frequent adverse effects associated with BPaLM include hepatotoxicity, cardiotoxicity, anemia, thrombocytopenia, and peripheral neuropathy, necessitating close monitoring [20].

### Study area

The MRJ has approximately 6.2 million inhabitants. Despite a relatively high Human Development Index (HDI) of 0.79 and an average monthly income of formal workers equivalent to 3.9 minimum wages, the city remains highly heterogeneous, with pronounced socioeconomic inequalities [9]. Around 22% of the population (~1.43 million people) live in favelas, and 31.4% have a monthly per capita income of up to half the minimum wage. Additionally, only 44.2% of the population is formally employed [21,22].

Moreover, Rio de Janeiro, the capital of the state with the third-highest incidence of drug-susceptible tuberculosis in Brazil, recorded the highest number of DR-TB cases nationwide in 2022. That year, the city accounted for approximately 70% of all DR-TB cases reported in the state of Rio de Janeiro [23].‌‌

In Brazil, between 2013 and 2022**,** drug-resistant tuberculosis cases initially showed a predominance of rifampicin resistance (40.5%), followed by multidrug resistance to first-line drugs (24.2%) and extensive drug resistance (1.4%). Mono-resistance and poly-resistance accounted for 24.2% and 7.7% of cases, respectively [24]. In contrast, data from the municipality of Rio de Janeiro analyzed in this study revealed distinct resistance patterns: rifampicin resistance was observed in 47.8% of cases, multidrug resistance in 15.7%, and extensive drug resistance in 0.5%. Mono-resistance and poly-resistance accounted for 22.1% and 13.7% of cases, respectively.

### Statistical analysis

The following demographic and epidemiological characteristics were considered independent variables: sex, race/skin color, education level, age group, type of resistance (primary or acquired), number of previous treatments, initial resistance pattern (monoresistance, polyresistance, MDR, or XDR-TB), and the presence of diseases and conditions associated with tuberculosis at the time of notification, including diabetes, AIDS, smoking, alcoholism, and illicit drug use. For these variables, “No” and “unknown” responses were combined. Despite representing distinct data entry conditions, this approach was adopted due to the high proportion of incomplete information and to ensure analytical feasibility.

Treatment regimens were classified into analytical categories based on their nomenclature and composition. The following categories were defined: MDR (multidrug-resistant tuberculosis, defined as resistance to at least rifampicin and isoniazid), MR (monoresistant tuberculosis, defined as resistance to a single first-line drug), individualized regimens (therapeutic approaches tailored to each patient based on prior treatment history and drug susceptibility testing results), SimpliciTB (the BPaMZ regimen, consisting of bedaquiline, pretomanid, moxifloxacin, and pyrazinamide), and others (less frequent regimens not classified in the previous categories). Second-line drug resistance was assessed by identifying patients with resistance to at least one second-line drug, including amikacin, fluoroquinolones, linezolid, and bedaquiline. Treatment duration was estimated as the interval between treatment initiation and the recorded outcome, considering all treatment outcomes.

Descriptive analyses of the independent variables were performed based on the outcomes using absolute and relative frequencies. Pearson’s chi-square test was used to test for significant differences between categories (p < 0.05).

Factors associated with unfavorable outcomes (treatment interruption, treatment failure, and death) were analyzed using a simple (unadjusted) multinomial logistic regression model. In this approach, each independent variable was analyzed separately, without simultaneous adjustment for other covariates. Odds ratios (ORs) and their respective 95% confidence intervals (95% CIs) were estimated. Treatment success was defined as the reference category for the outcome variable and compared with the other outcome categories (loss to follow-up, treatment failure, and death). All statistical analyses were performed using R software (version 4.4.2).

### Spatial analyses

To identify purely spatial clusters of drug-resistant tuberculosis (DR-TB) cases with unfavorable outcomes, we employed the Spatial Cluster Analysis using a discrete Poisson model (SCAN statistic), a method designed for count data. The SCAN method detects the most likely clusters that deviate from the null hypothesis of a random spatial distribution and estimates the relative risk for each identified cluster.

For this spatial cluster analysis, we incorporated data on the number of DR-TB cases with unfavorable outcomes, the at-risk population size, the scanning window dimensions, and the geographic coordinates of each neighborhood’s representative polygon center. To accurately represent neighborhoods’ distribution, the coordinates were adjusted to align with each neighborhood’s area of highest population density.

In this study, the maximum spatial cluster size was set at up to 20% of the population at risk. This threshold is commonly used in spatial epidemiology to balance sensitivity in cluster detection while avoiding excessively large clusters that may reduce epidemiological interpretability. The choice was also informed by the demographic characteristics of the study area. Specifically, the 20% threshold allowed the inclusion of the most populous neighborhoods in potential clusters, while preventing the identification of clusters encompassing a substantial proportion of the study area. Lower thresholds would limit the detection of clusters in high-density areas, whereas higher thresholds could generate overly large and less informative clusters.

Additionally, exploratory analyses using alternative spatial window sizes produced similar spatial patterns, supporting the robustness and adequacy of the 20% parameter for the analytical scale adopted in this study.

Following parameter definition, the spatial scan statistic applied a circular scanning window across the study region to detect areas with an excess of cases. Clusters with a high observed-to-expected case ratio were identified as the most likely using a likelihood ratio test. In addition to detecting spatial clusters, the method quantifies relative risk by comparing the incidence of cases within each window to that of the surrounding area. For example, a relative risk of 2.0 indicates that the population within the cluster has twice the risk of that in other areas, after adjusting for the underlying population distribution. A circular scanning window including up to 20% of the city’s total population was specified. Relative risks for statistically significant clusters (p < 0.05) were subsequently mapped. Spatial cluster analysis was conducted using SaTScan (version 9.6.1). Incidence rates were also analyzed at the neighborhood level to further explore the spatial distribution of drug-resistant tuberculosis (DR-TB) in the municipality. The resulting maps were generated using QGIS. The shapefile containing neighborhood boundaries for the city of Rio de Janeiro was obtained from the Municipal Government of Rio de Janeiro.

To enhance the interpretation of spatial findings in relation to individual-level results, we conducted a descriptive analysis comparing key clinical and epidemiological characteristics of patients residing within the identified clusters and those residing outside them.

### Ethical considerations

This study was approved by the Research Ethics Committee of the Instituto de Estudos em Saúde Coletiva at Universidade Federal do Rio de Janeiro (UFRJ) under the report number 6.424.714. The requirement for informed consent was waived due to the use of anonymized secondary data and the minimal risk posed to participants.

## Results

Between 2015 and 2022, a total of 972 patients residing in the municipality of Rio de Janeiro were diagnosed with DR-TB and recorded in the health information system. Of these, 665 (68.4%) achieved treatment success, while 307 (31.6%) experienced unfavorable outcomes (Table 1).

**Table 1:** Characteristics of drug-resistant tuberculosis cases with unfavorable outcomes in the municipality of Rio de Janeiro, 2015 to 2022.

Characteristics	Loss to follow-up N = 197	Treatment failure N = 48	Deaths N = 62	P-value ^a^
**Gender**				0.003
Female	48 (24.4%)	13 (27.1%)	21 (33.9%)	
Male	149 (75.6%)	35 (72.9%)	41 (66.1%)	
**Race/skin color** ^**b**^				0.004
White	44 (23.4%)	8 (17.0%)	18 (29.5%)	
Black	69 (36.7%)	11 (23.4%)	20 (32.8%)	
Brown	75 (39.9%)	28 (59.6%)	23 (37.7%)	
**Schooling** ^**b**^				0.001
More than 8 years	47 (26.9%)	26 (57.8%)	17 (32.7%)	
Less than 8 years	128 (73.1%)	19 (42.2%)	35 (67.3%)	
**Age range**				0.001
20–39	108 (4.8%)	20 (41.7%)	16 (25.8%)	
40 –59	76 (38.6%)	21 (43.8%)	31 (50.0%)	
> 60	13 (6.6%)	7 (14.6%)	15 (24.2%)	
**Resistance type**				0.001
Primary	87 (44.2%)	25 (52.1%)	37 (59.7%)	
Acquired	110 (55.8%)	23 (47.9%)	25 (40.3%)	
**Prior treatments**				0.001
0	45 (22.8%)	12 (25.0%)	26 (41.9%)	
1	101 (51.3%)	23 (47.9%)	20 (32.3%)	
2 or more	51 (25.9%)	13 (27.1%)	16 (25.8%)	
**Drug resistance**				0.001
Mono/poly resistance	60 (30.5%)	8 (16.7%)	15 (24.2%)	
MDR/XDR-TB	137 (69.5%)	40 (83.3%)	47 (75.8%)	
**Aids**				0.002
No/ unknown	173 (87.8%)	42 (87.5%)	50 (80.6%)	
Yes	24 (12.2%)	6 (12.5%)	12 (19.4%)	
**Alcohol abuse**				0.001
No/ unknownsu	131 (66.5%)	40 (83.3%)	42 (67.7%)	
Yes	66 (33.5%)	8 (16.7%)	20 (32.3%)	
**Diabetes**				
No/ unknown	184 (93.4%)	37 (77.1%)	55 (88.7%)	0.001
Yes	13 (6.6%)	11 (22.9%)	7 (11.3%)	
**Smoking**				0.009
No/ unknown	131 (66.5%)	31 (64.6%)	39 (62.9%)	
Yes	66 (33.5%)	17 (35.4%)	23 (37.1%)	
**Illicit drug use**				0.001
No/ unknown	118 (59.9%)	39 (81.2%)	50 (80.6%)	
Yes	79 (40.1%)	9 (18.8%)	12 (19.4%)	

**Note:** ^a^ Pearson’s Chi-squared test; Fisher’s exact test. ^b^ Cases with unknown information were excluded. Missing data for schooling and race/skin color by outcome were: death (10/62 for schooling and 1/62 for race/skin color), treatment failure (3/48 and 1/48), and loss to follow-up (22/197 and 9/197), respectively. For comorbidities, the categories “no” and “unknown” were grouped; missing data by outcome: diabetes (124/197; 25/48; 36/62), smoking (99/197; 22/48; 24/62), AIDS (119/197; 30/48; 29/62), alcohol abuse (95/197; 32/48; 25/62), and illicit drug use (90/197; 31/48; 34/62), corresponding to loss to follow-up, treatment failure, and death, respectively.

Among patients with unfavorable outcomes, 197 (20.2%) discontinued treatment, 48 (4.9%) experienced therapeutic failure, and 62 (6.3%) died from the disease (Table 1).

Regarding treatment regimens, most patients received individualized regimens (45.3%), followed by MDR (24.6%), other regimens (20.4%), MR (8.2%), and SimpliciTB (1.5%). Treatment duration ranged from 252 to 902 days (median: 367 days; interquartile range: 252–547).

Resistance to at least one second-line drug was observed in 28.7% (n = 279) of patients, although information on second-line drug resistance was unavailable for 71.3% of cases. Resistance to at least one fluoroquinolone was identified in 4.4% (n = 43) of patients.

Regarding sociodemographic characteristics, males accounted for the majority of cases across all categories of unfavorable outcomes, particularly among those lost to follow-up (75.6%). Individuals classified as Brown also represented the highest proportion across the three outcomes, especially in treatment failure (59.6%) (Table 1).

Patients who experienced loss to follow-up or death more frequently had fewer than 8 years of education (73.1% and 67.3%, respectively), whereas those with treatment failure more often had higher educational attainment (57.8% with more than 8 years of schooling) (Table 1).

Adults aged 20–39 and 40–59 years comprised the largest proportions of all unfavorable outcomes. Among those lost to follow-up, the 20–39 years age group accounted for 54.8% of cases, while individuals aged 60 years or older represented 6.6%. In cases of treatment failure, the most frequent age groups were 40–59 years (43.8%) and 20–39 years (41.7%), whereas among deaths, 50.0% occurred in individuals aged 40–59 years (Table 1).

Primary resistance was more frequent in cases of treatment failure (52.1%) and death (59.7%), while acquired resistance was more frequent among cases of loss to follow-up (55.8%). Previous treatment was reported in 51.3% of patients lost to follow-up and 47.9% of those with treatment failure, whereas among deaths, a higher proportion had no history of prior treatment (41.9%) (Table 1).

Regarding the initial resistance pattern, MDR/XDR-TB accounted for the majority of cases across all unfavorable outcomes (69.5% among those lost to follow-up, 83.3% in treatment failure, and 75.8% among deaths) (Table 1).

Regarding comorbidities and drug use, AIDS was reported in 19.4% of deaths, compared with approximately 12.0% in other unfavorable outcomes. Diabetes was reported in 22.9% of treatment failure cases, 6.6% of those who lost to follow-up, and 11.3% of deaths. Smoking was reported in 33.5%, 22.9%, and 37.1% of cases of loss to follow-up, treatment failure, and death, respectively. Illicit drug use was reported in 40.0% of cases lost to follow-up, 18.8% of treatment failure cases, and 19.4% of deaths (Table 1).

Table 2 presents the results of the multinomial logistic regression analysis of factors associated with unfavorable treatment outcomes in drug-resistant tuberculosis.

**Table 2:** Factors associated with unfavorable treatment outcomes in drug-resistant tuberculosis cases in the Municipality of Rio de Janeiro, 2015–2022.

Characteristics	Loss of follow-up		Treatment failure		Deaths	
**OR**	**CI 95%**	**OR**	**CI95%**	**OR**	**CI95%**
**Gender**						
Female	Ref.					
Male	**1.9**	**1.3–2.7**	1.6	0.8–3.8	1.2	0.6–2.1
**Race/Skin color**						
White	Ref.					
Black	**2.0**	**1.3–3.1**	1.7	0.7–4.5	1.4	0.7–2.8
Brown	1.2	0.8–1.8	**2.5**	**1.1–5.5**	0.9	0.4–1.7
**Schooling**						
More than 8 years	Ref.					
Less than 8 years	**2.4**	**1.7–3.6**	0.6	0.3–1.2	1.9	1.0–3.4
**Age range**						
20 - 39	Ref.					
40 - 59	0.8	0.5–1.0	1.1	0.6–2.1	**2.1**	**1.1–3.9**
> 60	**0.4**	**0.2–0.7**	1.2	0.4–2.8	**3.1**	**1.5–6.4**
**Resistance**						
Primary	Ref.					
Acquired	**2.5**	**1.8–3.5**	1.8	1.0–3.3	1.4	0.7–2.3
**Drug resistance**						
Mono/poly resistance	Ref.					
MDR/XDR-TB	1.52	1.0–2.1	**3.3**	**1.5 - 7.2**	**2.1**	**1.1–3.8**
**Prior treatments**						
0	Ref.					
1	**2.2**	**1.5–3.3**	1.9	0.9–3.9	0.8	0.4–1.4
2 or more	**3.3**	**2.1–5.3**	**3.2**	**1.4–7.2**	1.8	0.9–3.5
**Alcohol abuse**						
No/ unknown	Ref.					
Yes	**2.6**	**1.8–3.7**	1.0	0.5–2.2	**2.4**	**1.4–4.3**
**Aids**						
No/ unknown	Ref.					
Yes	**1.9**	**1.1–3.1**	1.9	0.8–4.8	**3.2**	**1.6–6.5**
**Diabetes**						
No/ unknown	Ref.					
Yes	**0.4**	**0.2–0.7**	1.5	0.7–3.0	0.6	0.2–1.4
**Smoking**						
No/ unknown	Ref.					
Yes	**1.6**	**1.1–2.2**	1.72	0.9–3.2	1.9	1.0–3.1
**Illicit drug use**						
No/ unknown	Ref.					
Yes	**3.8**	**2.7–5.5**‌‌	1.3	0.6–2.8	1.4	0.7–2.6

Note: 95% CI = 95% Confidence Interval; OR = Odds Ratio; Ref. = Reference category, **Bold values indicate statistically significant odds ratios (95% CI).**

In the multinomial analysis, treatment interruption was associated with male sex, Black race/ethnicity, and AIDS comorbidity, and acquired resistance (OR= 2.5; 95% CI: 1.8–3.5), compared with treatment success. Lower educational attainment (<8 years of schooling) was associated with higher odds of treatment interruption (OR=2.4; 95% CI: 1.7–3.3), as were alcohol abuse (OR = 2.6; 95% CI: 1.8–3.7), smoking, and illicit drug use. A history of more than two previous treatments for drug-susceptible tuberculosis was also associated with higher odds of treatment interruption (OR = 3.3; 95% CI:2.1–5.3). Age ≥ 60 years (OR=0.4; 95% CI: 0.2–0.7) and diabetes were associated with lower odds of treatment interruption (OR=0.4; 95% CI: 0.2–0.7) (Table 2).

In the multinomial analysis, treatment failure was associated with Brown race/color, MDR/XDR-TB, and a history of multiple previous treatments for drug-susceptible tuberculosis, compared with treatment success (Table 2).

Death was associated with age ≥ 60 years, MDR/XDR-TB, alcohol use, and AIDS comorbidity, compared with treatment success (Table 2).

**Fig 1:**
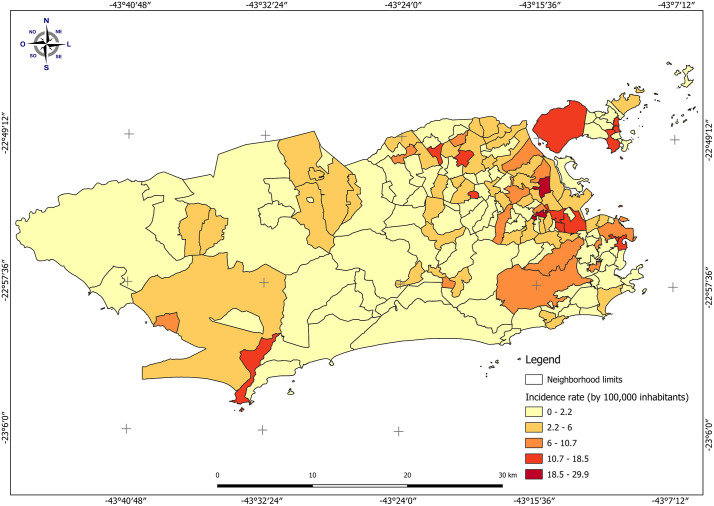
Spatial distribution of drug-resistant tuberculosis cases by neighborhood in the municipality of Rio de Janeiro, Brazil, from 2015 to 2022. Shapefile publicly available source: Data.Rio – Neighborhood boundary.

The spatial distribution of DR-TB incidence showed higher rates in neighborhoods located in the North Zone of Rio de Janeiro (Fig 1).

**Fig 2:**
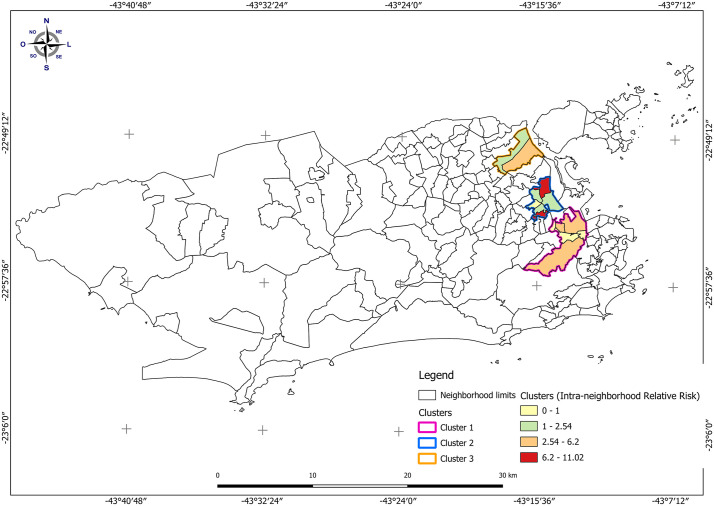
Spatial distribution of high-risk clusters for unfavorable outcomes of drug-resistant tuberculosis in the Municipality of Rio de Janeiro, Brazil, 2015–2022. Shapefile publicly available source: Data.Rio – Neighborhood boundary.

Three spatial clusters of unfavorable outcomes in DR-TB cases were identified in the North Zone of the city (Fig 2). The most likely cluster comprised five neighborhoods, with an estimated population of 225,537 inhabitants and a relative risk of 3.7 (Table 3).

**Table 3:** Characteristics of high-risk clusters for unfavorable outcomes of drug-resistant tuberculosis cases by neighborhood in the Municipality of Rio de Janeiro, Brazil.

Clusters/ Neighborhoods	Population	Cases	Incidence*	RR	LR	P-value
Maracanã, Mangueira, Praça da Bandeira, São Cristóvão e Tijuca	225.537	34	15.0	3.69	18.415	< 0.01
Manguinhos, Jacarezinho, Jacaré, Higienópolis e Bonsucesso	110.709	19	17.1	4.03	11.822	< 0.01
Penha e Penha Circular	95.241	14	14.7	3.40	7.061	0.02

Note: LR (Likelihood Ratio); RR (Relative Risk); Incidence per 100.000 inhabitants.

The secondary clusters collectively encompassed a population of 205,950 inhabitants, corresponding to 3.3% of the population of Rio de Janeiro, with relative risks of 4.0 and 3.4. The neighborhoods of Bonsucesso and Jacaré showed relative risks ranging from 6.2 to 11.0 (Table 3; Fig. 2).

We compared the clinical and epidemiological profiles of all patients with drug-resistant tuberculosis (n = 972) residing within (n = 190) and outside (n = 782) the detected clusters. Patients residing within clusters had significantly higher proportions of treatment abandonment (29% vs. 18%, p = 0.008), MDR/XDR-TB (74% vs. 62%, p = 0.001), and lower levels of schooling (67% vs. 54% with less than 8 years of education, p = 0.003). No statistically significant differences were observed for age, sex, race/color, type of resistance, number of previous treatments, or comorbidities (Table 4).‌‌

**Table 4:** Clinical and epidemiological characteristics of patients with drug-resistant tuberculosis, according to residence within spatial clusters of unfavorable treatment outcomes. Rio de Janeiro, 2015–2022.

Characteristics	Outside clusters n = 782	Within clusters n = 190	p-value^2^
Age (years)^1^	41 (30, 54)	42 (30, 53)	> 0.9
Gender			0.073
Female	259 (33%)	76 (40%)	
Male	523 (67%)	114 (60%)	
Race/Skin color			0.2
White	223 (29%)	54 (29%)	
Black	347 (45%)	71 (39%)	
Brown	201 (26%)	59 (32%)	
Schooling			0.003
More than 8 years	329 (46%)	59 (33%)	
Less than 8 years	391 (54%)	118 (67%)	
Treatment outcome			0.008
Loss to follow-up	142 (18%)	55 (29%)	
Treatment failure	41 (5.2%)	7 (3.7%)	
Death	49 (6.3%)	13 (6.8%)	
Treatment success	550 (70%)	115 (61%)	
Resistance type			>0.9
Acquired	305 (39%)	75 (39%)	
Primary	477 (61%)	115 (61%)	
Drug resistance			0.001
MDR/XDR-TB	482 (62%)	141 (74%)	
Mono/poly resistance	300 (38%)	49 (26%)	
Age range			0.8
20–39	359 (46%)	84 (44%)	
40 - 59	320 (41%)	83 44%)	
≥ 60	103 (13%)	23 (12%)	
Prior Treatment			0.8
0	293 (37%)	74 (39%)	
1	344 (44%)	84 (44%)	
2 or more	145 (19%)	32 (17%)	
Aids			0.052
No/Unknow	322 (81%)	89 (89%)	
Yes	77 (19%)	11 (11%)	
Diabetes			0.4
No/Unknow	311 (73%)	79 (77%)	
Yes	117 (27%)	24 (23%)	
Alcohol abuse			0.2
No/Unknow	295 (65%)	69 (59%)	
Yes	156 (35%)	47 (41%)	
Smoking			0.3
No/Unknow	274 (57%)	64 (52%)	
Yes	207 (43%)	60 (48%)	
Illicit drug use			0.5
No/Unknow	289 (65%)	69 (62%)	
Yes	156 (35%)	43 (38%)	

^1^Median (IQR); n (%); ^2^ Wilcoxon rank sum test; Pearson’s Chi-squared test.

## Discussion

The findings of this study indicate a high proportion of unfavorable treatment outcomes among patients with DR-TB, exceeding 30%. These results are consistent with previous studies conducted in Rio de Janeiro [3,8] and Brazil [4], reinforcing ongoing challenges in the management of DR-TB.

They also highlight the need for strengthened investment in DR-TB care and surveillance strategies tailored to the disease’s characteristics and risk factors, aiming to achieve the WHO treatment success target of at least 75% [19]. In this context, strengthening adherence support strategies – such as directly observed therapy, active follow-up of patients at higher risk of treatment interruption, and integration with substance use care services, such as mental health and addiction support programs – may be essential to improve treatment outcomes.

In this study, unfavorable outcomes were more frequent among men, young adults, Black or Brown individuals, and those with lower educational attainment, underscoring the well-established association between tuberculosis and social vulnerability. Similar patterns have been reported in other high-burden settings, where socioeconomic disadvantages and barriers to healthcare access contribute to poorer DR-TB outcomes.

The burden of tuberculosis in socially vulnerable populations is a well-established feature of drug-susceptible tuberculosis and is further amplified in drug-resistant forms, leading to a higher risk of unfavorable outcomes [8,25].

Evidence from a cohort study conducted in India among DR-TB patients highlights the impact of social, psychological, and economic support on treatment outcomes. Patients who received such support showed higher treatment success rates compared to those who did not (65.0% vs. 46.0%; p = 0.034), with reductions in both mortality and loss to follow-up [26].

These findings reinforce the importance of socioeconomic conditions in shaping both disease occurrence and treatment outcomes and support the implementation of patient-centered interventions to improve adherence and DR-TB outcomes.

These findings highlight the role of socioeconomic conditions in both disease occurrence and treatment outcomes and support the implementation of patient-centered interventions, including strengthened adherence support and improved access to coordinated care, to enhance DR-TB treatment outcomes.

Our findings show differences in sociodemographic and epidemiological characteristics across distinct unfavorable outcomes. Black race/skin color, lower education attainment, and male sex were associated with treatment discontinuation, consistent with the well-established relationship between social vulnerability and loss to follow-up. These findings are in agreement with a recent systematic review of national studies [27].

Similarly, acquired resistance, multiple prior treatments, smoking, abusive alcohol and illicit drug use, and AIDS diagnosis were associated with increased odds of loss to follow-up, consistent with previous studies [4,28]. These findings highlight the combined influence of clinical, behavioral, and socioeconomic factors on DR-TB treatment adherence.

Previous studies have reported a higher frequency of pulmonary tuberculosis and unfavorable treatment outcomes among men. These differences have been associated with a higher prevalence of risk behaviors, such as smoking and alcohol use, as well as lower use of health services and delays in seeking care, which may contribute to challenges in treatment adherence [29,30].

In contrast, higher educational attainment has been associated with greater engagement with health services and improved adherence to treatment protocols [31].

Alcohol and illicit drug use have been associated with poorer adherence and unfavorable DR-TB treatment outcomes [28]. Patients classified as lost to follow-up had higher odds of acquired resistance and prior treatment attempts, suggesting that adherence challenges may reflect both individual factors and limitations in sustaining long-term patient engagement within health services.

In DR-TB/HIV coinfection, treatment management is further complicated by barriers to integrated care and pharmacokinetic interactions between antiretrovirals and antituberculosis drugs. These interactions may increase the risk of toxicity and intolerance, potentially affecting treatment adherence and outcomes [32].

Diabetes and older age were associated with lower odds of treatment interruption due to loss to follow-up. Although previous studies have reported a negative impact of diabetes on DR-TB outcomes [33], particularly regarding treatment failure and mortality [28,34], our findings suggest a potential protective association between diabetes and treatment retention. This may be related to the more frequent contact with primary care services required for diabetes management, which can facilitate healthcare engagement and community care [35].

Patients over 60 years old showed a lower likelihood of treatment interruption, consistent with Viana et al. (2018) [36], who also reported old age as a protective factor against DR-TB treatment discontinuation. This pattern may be related to more frequent use of healthcare services and closer clinical follow-up among older individuals, which can facilitate treatment adherence [36].

Regarding treatment failure, Brown skin color, history of more than two previous treatments, and an MDR/XDR-TB resistance pattern were identified as associated factors. Previous incomplete or inadequate treatment, delays in identifying drug resistance, and extensive drug resistance may contribute to an increased risk of treatment failure.

Studies specifically evaluating risk factors for treatment failure remain limited, as most analyses address unfavorable DR-TB collectively (death, treatment failure, and treatment interruption). A national study of DR-TB cases by Bartholomay *et al.* (2021) [4] identified the use of more than one injectable drug, bilateral lung involvement, and clinical deterioration during treatment (including symptoms, imaging, and laboratory findings) as risk factors for treatment failure.

In fatal outcomes, advanced age, alcohol use, AIDS, and extensive drug resistance were identified as associated factors. These findings are consistent with previous studies, which have highlighted the role of immunosuppression, comorbidities, and treatment-related challenges in increasing the risk of death among DR-TB patients [4,26,37,38].

The spatial distribution of DR-TB revealed clusters of unfavorable outcomes concentrated in the Northern Zone of Rio de Janeiro. This region exhibits an overlap of neighborhoods with both high DR-TB incidence and increased risks of unfavorable outcomes. These patterns may indicate challenges in the organization and delivery of care, highlighting the need for further investigation of local programmatic factors. Strengthening local-scale surveillance and developing patient-centered interventions are essential for improving treatment adherence and reducing DR-TB mortality.

These patterns may indicate challenges in the organization and delivery of care, highlighting the need for further investigation of local programmatic factors. Strengthening local surveillance and implementing targeted, patient-centered interventions in these areas may be important to improve treatment adherence and reduce DR-TB mortality.

When integrating individual-level and spatial findings, the comparison between patients residing inside and outside the identified clusters provides additional insight. Individuals living within clusters of unfavorable outcomes showed higher proportions of treatment abandonment, MDR/XDR-TB, and lower educational attainment compared to those living outside these areas. These findings suggest that the spatial concentration of unfavorable outcomes reflects the aggregation of individual-level vulnerabilities within specific territories, reinforcing the importance of addressing both individual and area-level determinants to improve DR-TB outcomes.

This pattern is consistent with the social context of the Northern Zone of Rio de Janeiro, which is marked by significant inequality and a high concentration of densely populated favelas. These areas face inadequate housing and sanitation conditions, as well as barriers related to public security and access to healthcare services [39,40], which may contribute to both ongoing transmission and challenges in treatment and surveillance, particularly for DR-TB.

Identifying areas with higher DR-TB and unfavorable outcomes is essential to guide targeted public health actions, including the prioritization of resources, strengthening of local health services, and development of context-specific interventions to improve treatment outcomes.

This study presents additional limitations beyond those inherent in observational designs based on secondary data. In the municipality of Rio de Janeiro, despite an increase over the past 10 years, the proportion of new cases of drug-susceptible tuberculosis with laboratory confirmation has remained below 80%, which may indicate an underreporting of DR-TB cases. Similarly, the performance of culture tests in retreatment cases in Rio de Janeiro was below 70% during the same period [41], suggesting underreporting of cases. Furthermore, the study period includes the most affected years by the COVID-19 pandemic, which may have contributed to DR-TB underreporting due to reduced healthcare-seeking behavior, limited access to diagnostic and drug susceptibility testing, and disruptions in surveillance and reporting practices. Depending on its magnitude, this underreporting could affect the results of this study by compromising the representativeness of the DR-TB cases registered in the SITE-TB database.

Another potential limitation is the recording of comorbidities (AIDS and diabetes) and other associated conditions (alcohol use, illicit drug use, and smoking) in the notification system. The combination of “No” and “unknown” categories for comorbidity variables may have introduced non-differential misclassification, potentially attenuating the observed associations. As a result, the reported estimates should be interpreted as conservative, and the true magnitude of the associations may be underestimated.

Another potential classification bias may arise from inaccurate information on patients’ neighborhood of residence, particularly in slum areas, where unstable housing or informal addresses may affect the identification of spatial clusters. This may affect the accuracy of geographic risk estimates and influence the interpretation of high-risk areas.

Regarding the spatial scan statistics used in our study, a methodological limitation should be explicitly considered. The identifying clusters were based exclusively on the distribution of cases and the population at risk, without incorporating contextual variables. Consequently, although the method can detect spatial patterns of unfavorable outcomes, it does not account for socioeconomic, environmental, or health system factors that may underlie these patterns. Therefore, the findings should be viewed as hypothesis-generating rather than explanatory. Future studies using complementary analytical approaches, such as spatial regression models or multilevel analyses, are needed to investigate the role of contextual factors in shaping the observed spatial patterns.

Despite the limitations of this study, the findings support the strengthening of DR-TB management and surveillance through targeted interventions in high-risk areas, particularly by expanding directly observed therapy and reinforcing the role of Family Health Teams.

Adherence support should extend beyond medication supervision to include patient education, counseling, and coordinated care involving patients, healthcare providers, and the community [42]. Such approaches may help address the complex challenges of DR-TB management, particularly in socially vulnerable populations.

Access to economic support programs may play an important role in improving DR-TB outcomes among socially vulnerable populations. A cohort study in India demonstrated that patients receiving social, psychological, and economic support had higher treatment success rates and lower mortality and loss to follow-up [26]. In Brazil, the conditional cash transfer program Bolsa Família has been associated with reduced tuberculosis incidence and mortality, particularly among populations in extreme poverty [43].

Beyond Bolsa Família, since 2022, DR-TB patients in the state of Rio de Janeiro have been eligible to receive food assistance during treatment [44]. This underscores the importance of coordinated actions between health and social protection sectors to ensure access to such programs, particularly in areas with clusters of unfavorable outcomes.

Finally, effective DR-TB care depends on coordination between specialized health services and primary care. Strengthening integration and information exchange across these levels may improve continuity of care, enhance surveillance, and reduce unfavorable outcomes, especially in socioeconomically vulnerable settings.

## Conclusion

This study demonstrates that unfavorable DR-TB outcomes are not randomly distributed but spatially concentrated in socially vulnerable areas and associated with individual-level social and clinical factors. By integrating spatial and individual-level analyses, it provides evidence that the accumulation of vulnerabilities within specific urban territories contributes to poor treatment outcomes. These findings support the implementation of targeted, place-based interventions, including strengthened adherence support, improved integration between primary care and specialized services, and expanded access to social protection programs in high-risk areas. Although based on data from Rio de Janeiro, these results may inform DR-TB control strategies in other large urban settings in Latin America facing similar social and epidemiological challenges.
